# Antibiotic exposure for culture-negative early-onset sepsis in late-preterm and term newborns: an international study

**DOI:** 10.1038/s41390-024-03532-6

**Published:** 2024-09-17

**Authors:** Varvara Dimopoulou, Claus Klingenberg, Lars Navér, Viveka Nordberg, Alberto Berardi, Salhab el Helou, Gerhard Fusch, Joseph M. Bliss, Dirk Lehnick, Nicholas Guerina, Joanna Seliga-Siwecka, Pierre Maton, Donatienne Lagae, Judit Mari, Jan Janota, Philipp K. A. Agyeman, Riccardo Pfister, Giuseppe Latorre, Gianfranco Maffei, Nicola Laforgia, Enikő Mózes, Ketil Størdal, Tobias Strunk, Martin Stocker, Eric Giannoni, the AENEAS Study Group, Capretti Maria Grazia, Ceccoli Martina, De Angelis Morena, Drimaco Pietro, Eap Khalyane, el Helou Zoe, Esmaeilizand Rana, Foglianese Alessandra, Geraci Carmelo, Grochowski Bartłomiej, Håkansson Stellan, Kaur Sharandeep, Kollegger Anne-Louise, Oldendorff Frida, Rizzo Vittoria, Arild E. Rønnestad, Shrestha Damber, Stensvold Hans Jørgen, Trefny Martin, Zilinska Kristyna, Zwijacz Aleksandra

**Affiliations:** 1 Clinic of Neonatology, Department Mother-Woman-Child, Lausanne University Hospital and University of Lausanne, Lausanne, Switzerland; 2 Research Group for Child and Adolescent Health, Faculty of Health Sciences, UiT-The Arctic University of Norway, Tromsø, Norway; 3 Department of Pediatrics and Adolescence Medicine, University Hospital of North Norway, Tromsø, Norway; 4 Department of Neonatology, Karolinska University Hospital and Department of Clinical Science, Intervention and Technology, Karolinska Institutet, Stockholm, Sweden; 5 Neonatal Intensive Care Unit, Mother and Child Department, Policlinico University Hospital, Modena, Italy; 6 Division of Neonatology, Department of Pediatrics, McMaster Children’s Hospital, McMaster University, Hamilton Health Sciences, Hamilton, Ontario, ON Canada; 7 Division of Neonatology, Department of Pediatrics, University of Rochester Medical Center, Rochester, NY USA; 8 Biostatistics and Methodology, CTU-CS, Faculty of Health Sciences and Medicine, University of Lucerne, Lucerne, Switzerland; 9 Department of Pediatrics, Women & Infants Hospital of Rhode Island, Warren Alpert Medical School of Brown University, Providence, RI USA; 10 Department of Neonatology and Neonatal Intensive Care, Medical University of Warsaw, Warsaw, Poland; 11 Neonatal Service, CHC-Montlegia Clinic, CHC Health Group, Liège, Belgium; 12 Neonatology and Neonatal Intensive Care Unit, CHIREC-Delta Hospital, Brussels, Belgium; 13 Department of Paediatrics, University of Szeged, Szeged, Hungary; 14 Neonatal Unit, Department of Obstetrics and Gynecology, Motol University Hospital Prague, Prague, Czech Republic; 15 Department of Pathological Physiology, 1st Medical School, Charles University Prague, Prague, Czech Republic; 16 Department of Neonatology, Thomayer University Hospital Prague, Prague, Czech Republic; 17 Division of Pediatric Infectious Disease, Department of Pediatrics, Inselspital, Bern University Hospital, University of Bern, Bern, Switzerland; 18 Neonatology and Paediatric Intensive Care Unit, Geneva University Hospitals and Geneva University, Geneva, Switzerland; 19 Neonatology and Neonatal Intensive Care Unit, Ecclesiastical General Hospital F. Miulli, Acquaviva delle Fonti, Italy; 20 Neonatology and Neonatal Intensive Care Unit, Policlinico Riuniti Foggia, Foggia, Italy; 21 Neonatology and Neonatal Intensive Care Unit, University of Bari, Bari, Italy; 22 Perinatal Intensive Care Unit, Department of Obstetrics and Gynaecology, Semmelweis University, Budapest, Hungary; 23 Department of Pediatric Research, Institute of Clinical Medicine, University of Oslo and Oslo University Hospital, Oslo, Norway; 24 Neonatal Directorate, Child and Adolescent Health Service, King Edward Memorial Hospital, Perth, WA Australia; 25 Department of Pediatrics, Children’s Hospital Lucerne, Lucerne, Switzerland; 26 Neonatal Intensive Care Unit, IRCCS Azienda Ospedaliero-Universitaria di Bologna, Bologna, Italy; 27 Neonatal Intensive Care Unit, Azienda Ospedaliero-Universitaria di Modena, Modena, Italy; 28 Neonatal Intensive Care Unit, Maggiore Hospital, Bologna, Italy; 29 University of Lausanne, Lausanne, Switzerland; 30 Neonatologia e Terapia Intensiva Neonatale, University of Bari, Bari, Italy; 31 Policlinico Riuniti di Foggia, Foggia, Italy; 32 The Swedish Neonatal Quality Register, Stockholm, Sweden and Department of Clinical Sciences, Pediatrics, Umeå University, Umeå, Sweden; 33 Department of Pediatrics, St Catharines General Hospital, Niagara Health, Niagara, ON Canada; 34 MACCS Université Catholique de Louvain, Louvain, Belgium; 35 Terapia Intensiva Neonatale Ospedale M.Bufalini Cesena, Cesena, Italy; 36 Institute for clinical medicine, University of Oslo, Oslo, Norway; 37 Department of Neonatal Intensive Care, Clinic of Paediatric and Adolescent Medicine, Oslo University Hospital, Oslo, Norway; 38 Department of Obstetrics and Gynecology, Neonatal unit, Motol University Hospital and Second Faculty of Medicine, Charles University, Prague, Czech Republic; 39 Department of Neonatology, Thomayer Hospital Prague, Prague, Czech Republic

## Abstract

**Background:**

Early-life antibiotic exposure is disproportionately high compared to the burden of culture-proven early-onset sepsis (CP-EOS). We assessed the contribution of culture-negative cases to the overall antibiotic exposure in the first postnatal week.

**Methods:**

We conducted a retrospective analysis across eleven countries in Europe, North America, and Australia. All late-preterm and term infants born between 2014 and 2018 who received intravenous antibiotics during the first postnatal week were classified as culture-negative cases treated for ≥5 days (CN ≥ 5d), culture-negative cases treated for <5 days (CN < 5d), or CP-EOS cases.

**Results:**

Out of 757,979 infants, 21,703 (2.9%) received intravenous antibiotics. The number of infants classified as CN ≥ 5d, CN < 5d, and CP-EOS was 7996 (37%), 13,330 (61%), and 375 (1.7%). The incidence of CN ≥ 5d, CN < 5d, and CP-EOS was 10.6 (95% CI 10.3–10.8), 17.6 (95% CI 17.3–17.9), and 0.49 (95% CI 0.44–0.54) cases per 1000 livebirths. The median (IQR) number of antibiotic days administered for CN ≥ 5d, CN < 5d, and CP-EOS was 77 (77–78), 53 (52–53), and 5 (5-5) per 1000 livebirths.

**Conclusions:**

CN ≥ 5d substantially contributed to the overall antibiotic exposure, and was 21-fold more frequent than CP-EOS. Antimicrobial stewardship programs should focus on shortening antibiotic treatment for culture-negative cases.

**Impact:**

- In a study of 757,979 infants born in high-income countries, we report a presumed culture-negative early-onset sepsis incidence of 10.6/1000 livebirths with an associated antibiotic exposure of 77 antibiotic days per 1000 livebirths.
- This study sheds light on the major contribution of presumed culture-negative early-onset sepsis to early-life antibiotic exposure.
- Given the diagnostic uncertainty surrounding culture-negative early-onset sepsis, the low mortality rate, and the disproportionate antibiotic exposure associated with this condition, our study emphasizes the importance of targeting culture-negative early-onset sepsis in antimicrobial stewardship programs.

## Introduction

Up to 15% of late preterm and term newborns are started on antibiotics during the first postnatal week.^1–4^ The justification for this approach is that newborns presenting with non-specific symptoms could have early-onset sepsis (EOS), requiring immediate treatment with antibiotics to prevent severe disease, death, or disability.^5^ In a large international study on late-preterm and term newborns, we recently reported that for every 58 infants started on antibiotics, only one turned out to have culture-proven EOS (CP-EOS).^6^ Antibiotic treatment for at least 7 days is recommended for CP-EOS.^7^ In the majority of infants for whom antibiotics are started empirically and subsequently have negative cultures, treatment can be safely stopped after 36 to 48 h.^7^

As signs of infection are nonspecific and the performance of biomarkers is limited, physicians may still be concerned for EOS despite the absence of a positive blood or cerebrospinal fluid (CSF) culture.^8–10^ These cases, often coined as culture-negative EOS (CN-EOS) may drive prolonged antibiotic treatment.^10^ Given the potentially detrimental impact of early-life antibiotic overuse, it is essential to minimize unnecessary exposure to antibiotics in newborns.^11–14^ Previous studies have underscored the difficulty in interpreting guidelines for discontinuing empiric antibiotic therapy in case of negative blood culture, often resulting in subjective decisions made independently of culture results.^15–23^ However, large international datasets reflecting a broad range of guidelines and practices are lacking. We hypothesized that presumed CN-EOS is a major driver of antibiotic exposure in late-preterm and term infants, representing a priority target for antimicrobial stewardship (AMS) interventions. Using a large international dataset of late-preterm and term infants born in high-income settings, we determined the incidence of presumed CN-EOS and related antibiotic exposure during the first postnatal week.

## Methods

### Study design

We conducted a secondary analysis of the Antibiotic Exposure for Suspected Neonatal Early-Onset Sepsis (AENEAS) study.^6^ Thirteen networks from 11 high-income countries in Europe, North America, and Australia participated. A network was defined by the adoption of a unified strategy for preventing and managing suspected EOS, coupled with the capacity to provide data on a minimum of 25,000 livebirths over the 5-year study period.^6^ We included all infants born alive in one of the participating networks at a gestational age ≥34 weeks between January 1st, 2014, and December 31st, 2018, and collected data on those who received intravenous antibiotics during the first postnatal week. Ethical approvals were obtained from the Swiss National Ethics Committee on human research and the ethics committees of all participating networks. The study adhered to the guidelines set forth by the Strengthening the Reporting of Observational Studies in Epidemiology for Newborn Infection (STROBE-NI).^24^

### Definitions

**Fig. 1:**
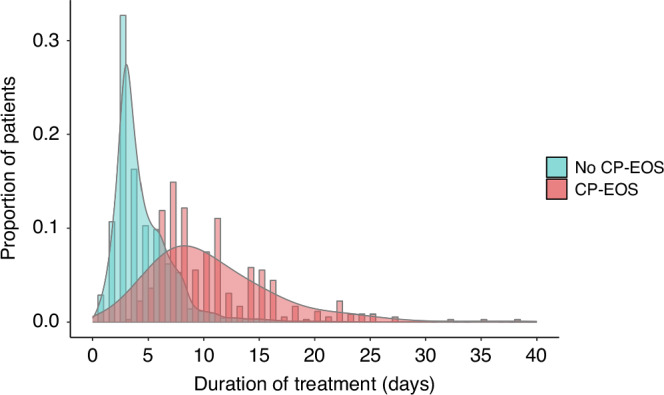
Duration of antibiotic treatment in infants with negative cultures and in infants with culture-proven early-onset sepsis. Histogram and density plot of the duration of antibiotic treatment for infants with negative cultures (No CP-EOS) and for infants with culture-proven early-onset sepsis (CP-EOS) in the whole cohort. Data is presented as the proportion of infants with No CP-EOS and with CP-EOS.

We defined CP-EOS by a positive blood and/or CSF culture within the first postnatal week. Contamination was defined by growth of bacteria typically regarded as contaminants (e.g., diphtheroids or *Micrococcus* species) or when cultures were deemed as contaminated by clinicians, leading to a decision to treat with antibiotics for less than 5 days. Infants with growth of coagulase-negative staphylococci (CoNS) in blood or CSF cultures and who received antibiotic therapy of more than 5 days were classified as having CP-EOS.^6^ Infants who received antibiotic therapy during the first postnatal week but who did not have a positive blood or CSF culture were classified in two groups depending on duration of antibiotic treatment. The group treated for ≥5 days was classified as CN ≥ 5d, corresponding to presumed CN-EOS. The group treated for <5 days was classified as CN < 5d, corresponding to so-called “ruled-out” sepsis.^25^ The decision to choose a cutoff at 5 days was made based on the literature and after visual inspection of histograms representing the duration of antibiotic treatment in culture-negative cases (Fig. 1 and Supplementary Fig. 1).^9,16,26^

Neonatal death was defined as either death before discharge or death occurring before 28 days for patients hospitalized beyond 28 days.^6^ Mortality associated with CP-EOS was defined as death occurring within 28 days after a positive blood and/or CSF culture. Mortality associated with CN ≥ 5d and CN < 5d, was defined by death within 28 days after the initiation of antibiotic treatment.

### Measurements

The primary outcome was to quantify the incidence of presumed CN-EOS and related antibiotic exposure and compare it with CP-EOS and ruled-out sepsis. The secondary outcomes were to describe the mortality within each group and network and analyze the distribution of the duration of antibiotic treatment among infants with and without CP-EOS without using specific duration cutoffs for culture-negative cases.

### Statistical analysis

The incidence of CN ≥ 5d, CN < 5d and CP-EOS was defined as the rate among all live-born neonates. Treatment duration was defined as calendar days with at least one dose of antibiotics. Antibiotic exposure was calculated as the sum of antibiotic days for each treated newborn divided by the number of livebirths and was reported per 1’000 livebirths.

Descriptive statistics were reported as median and interquartile range (IQR) for continuous variables and as frequencies with 95% confidence intervals (CIs) for categorical variables. The Spearman correlation coefficient (R) was employed to evaluate correlations between quantitative metrics. A weighted correlation (Rw) was calculated to emphasize the varying importance of each data point in the analysis. The analyses were executed using R version 4.3.0 (R Project for Statistical Computing).

## Results

### Incidence of CN ≥ 5d, CN < 5d, and CP-EOS

**Fig. 2:**
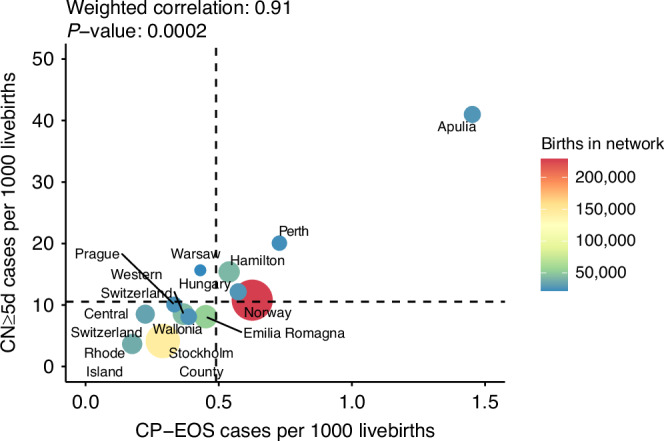
Relationship between the incidence of culture-negative cases with an antibiotic treatment of at least 5 days and the incidence culture-proven early-onset sepsis. Relationship between the incidence of culture-negative cases with an antibiotic treatment of at least five days (CN ≥ 5d) and the incidence of culture-proven early-onset sepsis (CP-EOS) cases in each network. The size of the bubbles represents the number of births. The dashed lines represent the median of the 13 networks.

Among the 21,703 infants started on intravenous antibiotics during the first postnatal week, 7996 (37%; 95% CI 36–38) were classified as CN ≥ 5d, 13,330 (61%; 95% CI 61–62) were classified as CN < 5d, and 375 had CP-EOS (1.7%; 95% CI, 1.6–1.9). Two patients with negative blood cultures were excluded due to missing data on duration of antibiotic treatment. The incidence of CN ≥ 5d, CN < 5d, and CP-EOS was 10.6 (95% CI 10.3–10.8), 17.6 (95% CI 17.3–17.9), and 0.49 (95% CI 0.44–0.54) cases per 1000 livebirths (Table 1). The incidence of CN ≥ 5d, CN < 5d, and CP-EOS ranged from 3.7 to 40.9 (11-fold variation), 2.1 to 103.7 (49-fold variation), and 0.18 to 1.5 (8-fold variation) per 1000 livebirths in different networks (Supplementary Fig. 2). CN ≥ 5d was 22 times more frequent than CP-EOS (range 14 to 38 in different networks). The incidence of CN ≥ 5d correlated positively with the incidence of CP-EOS in each network (R = 0.79, *P* = 0.002; Rw = 0.90, *P* = 0.0002) (Fig. 2).

**Table 1:** Main outcomes in each network

Network	Incidence^a^ *n* (per 1000 livebirths)			Mortality *n* (%)			Duration of antibiotic treatment (calendar days)			Antibiotic exposure (antibiotic days per 1000 livebirths)		
	CN ≥ 5d	CN < 5d	CP-EOS	CN ≥ 5d	CN < 5d	CP-EOS	CN ≥ 5d	CN < 5d	CP-EOS	CN ≥ 5d	CN < 5d	CP-EOS
Norway	2465 (10.8)	3159 (13.8)	143 (0.63)	21 (0.90)	58 (1.8)	3 (2.1)	6 (5–7)	3 (2–4)	8 (6–11)	68 (67–69)	41 (40–42)	6 (6–6)
Stockholm County	594 (4.1)	1065 (7.4)	42 (0.29)	12 (2.0)	18 (1.7)	2 (4.8)	6 (5–8)	3 (2–4)	9 (8–11)	30 (29–31)	21 (20–22)	3 (3–3)
Central Switzerland	303 (8.5)	634 (17.8)	8 (0.22)	2 (0.66)	5 (0.79)	0	6 (5–6)	3 (2–4)	10 (9–14)	53 (50–55)	52 (49–54)	2 (2–3)
Emilia Romagna	448 (8.1)	458 (8.3)	25 (0.45)	6 (1.3)	9 (1.9)	3 (12.0)	6 (5–8)	3 (3–4)	8 (7–14)	62 (60–64)	26 (25–28)	5 (4–5)
Western Switzerland	393 (8.5)	893 (19.3)	17 (0.37)	3 (0.76)	11 (1.2)	1 (5.9)	7 (6–8)	3 (3–3)	10 (8–14)	64 (62–67)	58 (56–60)	4 (4–5)
Hamilton^b^	685 (15.4)	1413 (31.8)	24 (0.54)	4 (0.58)	12 (0.85)	1 (4.2)	8 (7–8)	3 (3–4)	12 (9–15)	125 (122–128)	99 (96–101)	7 (7–8)
Rhode Island	146 (3.7)	810 (20.3)	7 (0.18)	0	4 (0.49)	0	7 (7–8)	3 (3–3)	12 (11–16)	27 (26–29)	58 (56–60)	2 (2–3)
Hungary	360 (12.1)	908 (30.6)	17 (0.57)	5 (1.4)	6 (0.66)	0	6 (5–8)	3 (2–3)	8 (6–11)	85 (82–89)	88 (85–91)	6 (5–7)
Apulia^b^	1213 (40.9)	63 (2.1)	43 (1.5)	2 (0.17)	2 (3.2)	0	8 (6–10)	4 (4–4)	11 (8–15)	362 (357–368)	8 (7–9)	17 (16–19)
Wallonia	230 (8.1)	475 (16.7)	11 (0.39)	2 (0.87)	4 (0.84)	0	6 (5–8)	4 (3–4)	11 (11–15)	54 (52–57)	60 (58–63)	5 (4–6)
Prague	272 (10.1)	229 (8.5)	9 (0.33)	0	1 (0.44)	1 (11.1)	6 (5–7)	3 (1–3)	7 (7–14)	65 (62–68)	20 (19–22)	3 (3–4)
Perth	524 (20.1)	2706 (103.7)	19 (0.73)	4 (0.76)	16 (0.59)	0	6 (5–7)	3 (3–3)	9 (7–15)	174 (170–179)	308 (302–314)	9 (8–10)
Warsaw	363 (15.7)	517 (22.3)	10 (0.43)	4 (1.1)	16 (3.1)	1 (10.0)	6 (6–8)	3 (3–4)	12 (8–11)	118 (114–122)	74 (70–77)	5 (4–6)
All networks	7996 (10.6)	13330 (17.6)	375 (0.49)	65 (0.81)	162 (1.2)	12 (3.2)	6 (5–8)	3 (3–4)	9 (7–14)	77 (77–78)	53 (52–53)	5 (5–5)

Categorical variables are presented as frequencies (%) and continuous variables as median (IQR). Column percentages are presented; percentages are based on available data for each variable.

^a^Data are missing for 2 patients with no culture-proven sepsis.

^b^Data on the duration of antibiotic treatment is missing for 1 patient.

### Antibiotic exposure in CN ≥ 5d, CN < 5d, and CP-EOS

**Fig. 3:**
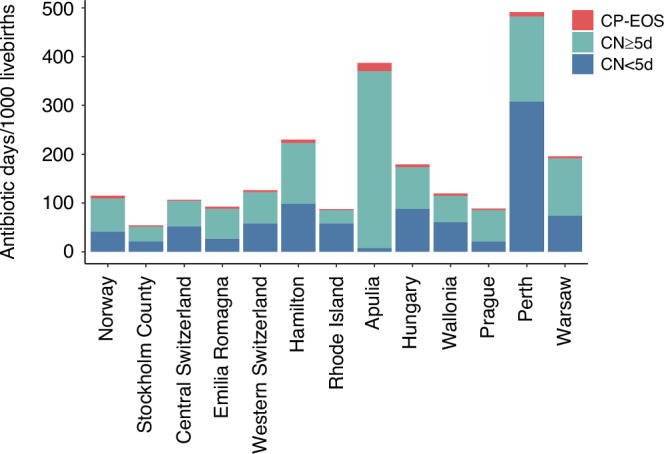
Antibiotic exposure for culture-negative cases with antibiotic treatment for at least 5 days, culture-negative cases with antibiotic treatment for less than 5 days and for culture-proven early-onset sepsis. Number of antibiotic days per 1000 livebirths in each network for culture-negative cases treated for at least 5 days (CN ≥ 5d), culture-negative cases treated shorter than 5 days (CN < 5d), and culture-proven early-onset sepsis (CP-EOS) cases.

The median (IQR) duration of treatment was 6 (5–8) days for CN ≥ 5d, 3 (3-4) days for CN<5d, and 9 (7–14) days for CP-EOS. The median (IQR) number of antibiotic days administered per 1000 livebirths was 77 (77–78) days for CN ≥ 5d, 53 (52–53) days for CN < 5d, and 5 (5–5) days for CP-EOS. Across different networks, the median number of antibiotic days administered per 1000 livebirths ranged from 27 to 362 in CN ≥ 5d (13-fold variation), from 8 to 307 days in CN < 5d (38-fold variation), and from 2 to 17 in CP-EOS (9-fold variation) (Fig. 3).

### Changes over time

Over time, when analyzing data without Norway (providing only 2015–2018 data), the incidence of CN ≥ 5d decreased from 11.9 (95% CI 11.3–12.6) in 2014 to 9.2 (95% CI 8.7–9.8) in 2018, and the incidence of CN < 5d decreased from 21.8 (95% CI 20.9–22.7) in 2014 to 17.4 (95% CI 16.6–18.2) in 2018 (Supplementary Fig. 3). Similarly, the median (IQR) number of antibiotic days administered for CN ≥ 5d and CN < 5d decreased over time, from 76 (70–83) to 60 days (54–75), and from 60 (55–66) to 50 days (45–55) days per 1000 livebirths (Supplementary Fig. 4).

### Mortality associated with CN ≥ 5d, CN < 5d, and CP-EOS

All-cause mortality rate was 0.81% (95% CI 0.62–1.0) for CN ≥ 5d, 1.2% (95% CI 1.0–1.4) for CN < 5d, and 3.2% (95% CI 1.7–5.5) for CP-EOS. The median (IQR) postnatal age at death was 10 (5–21) days for CN ≥ 5d, 2 (1–4) days for CN < 5d, and 4 (2–10) days for CP-EOS. Data on cause of death was available for 66% (157/239) of fatal cases (Norway could not provide the data). In culture-negative cases, CN ≥ 5d and CN < 5d, no deaths were attributed to sepsis, with most of them attributed to malformations (20/44, 46% and 59/104, 57%), followed by perinatal asphyxia (15/44, 34% and 28/104, 27%). In CP-EOS, all deaths were related to sepsis (6/9, 67% directly related and 3/9, 33% indirectly related), and most deaths were attributed to respiratory and/or cardiocirculatory failure (7/9, 78%), with 2/9 (22%) attributed to malformations.

## Discussion

In this large international study, CN ≥ 5d accounted for over half of the total number of antibiotic days administered to late-preterm and term newborns in the first postnatal week and was not associated with sepsis-related mortality. This suggests that presumed CN-EOS is a major driver of antibiotic use, disproportionately contributing to the burden of antibiotic exposure in early life.

CN-EOS is a condition where the treating physician decides not to discontinue empirically started antibiotics despite negative cultures. This practice is supported by the arguments that the sensitivity of blood and CSF cultures is below 100%, not all invasive infections are associated with bacteremia (e.g., pneumonia), and that current biomarkers have insufficient performance to unequivocally rule out a bacterial infection.^20^ Criteria used to define CN-EOS vary widely in the literature and are generally based on the physicians’ decision to treat a patient for at least 3, 5 or 7 days.^9,16–20^ Clinical signs and biomarker levels are frequently, but inconsistently used as additional diagnostic criteria.^10,23^ A single-center study identified that physicians tend to continue antibiotics for more than 3 days despite negative cultures in the presence of elevated C-reactive protein levels, abnormal white blood cell count values, need for vasopressors and/or mechanical ventilation.^23^ However, several national guidelines recommend that abnormal laboratory tests alone should not justify prolonged empiric antibiotic therapy without evidence of site-specific infection or critical illness and emphasize the importance of stopping antibiotics within 36–72 h in the absence of confirmed bacterial infection.^7,27–29^

The analysis of distribution of duration of antibiotic treatment among infants with and without CP-EOS provides a better understanding of the approaches used in daily clinical practice. In the entire cohort, the length of treatment appeared as a continuum, with no specific cutoff and considerable overlap between patients with and without CP-EOS. However, in four out of 13 networks, we found a bimodal distribution in the duration of treatment in patients without CP-EOS, suggesting that patients in whom EOS was ruled out were treated 3 to 4 days, whereas patients with presumed CN-EOS were treated 5 to 10 days.

In our study, the incidence of CN ≥ 5d was 10.6 per 1000 livebirths, which is 21 times more frequent than CP-EOS. In contrast, other studies with an incidence of CP-EOS ranging from 0.34 to 1.6 per 1000 livebirths reported an incidence of presumed CN-EOS of 4.2 to 9.3 per 1000 livebirths in late-preterm and term newborns.^16,19–21^ The lower CN-EOS/CP-EOS ratio of 6–16 in those studies could be related to the fact that they did not include all late-preterm and term infants and used different criteria to diagnose CN-EOS and CP-EOS.^9^ An estimation based on blood culture sensitivity and a mathematical equation suggests that CN-EOS should be up to 10-fold less frequent than CP-EOS.^10^ When 1 ml of blood is inoculated according to current recommendations, cultures can detect up to 98% of a bacterial load of at least 4 CFU/ml, and up to 95% of low-colony-count bacteremia.^7,26,30^ As the proportion of blood cultures with a minimum of 1 ml ranges from 64% to 93% in different studies,^31–33^ it is crucial to optimize blood culture collection technique to reduce the risk of false-negatives.^34,35^ Median time to positivity of blood cultures is 12–15 h, and 96% of the positive blood cultures are detected by 36 h in newborns.^36,37^ Therefore, blood cultures drawn adequately can be trusted if they remain negative after 24–36 h of sampling, and empirically started antibiotics can be safely stopped in most cases with negative cultures. Our data indicate that antibiotic treatment is often prolonged beyond 2 days in culture-negative cases, and that presumed CN-EOS is substantially overdiagnosed. The positive correlation between the incidence of CN ≥ 5d and CP-EOS in each network suggests that the attitude of physicians regarding prolonging duration of antibiotic treatment in neonates despite negative blood and CSF cultures might be driven by their local incidence of CP-EOS. It also suggests that all networks may have a potential to improve their strategies in diagnosing and treating CN- EOS. There were substantial variations regarding the guidelines used to prevent and treat EOS in different networks.^6^ However, we could not identify strategies associated with a lower incidence of CN-EOS. We speculate that the level of implementation of best practices to prevent EOS and promote rationale use of antibiotics could have had an important impact.^38^

All- cause mortality in the CN ≥ 5d group was 4.0-fold lower than in CP-EOS, and 1.5-fold lower than in the CN < 5d group. Moreover, deaths in the CN ≥ 5d group were not attributed to suspected infection, as fatal cases were associated with non-infectious conditions (perinatal asphyxia and congenital malformations). This is supported by recent literature showing that CN-EOS is associated with a lower risk of death compared to CP-EOS.^39–41^

The fear of inadequately treating a patient with presumed CN-EOS and the perceived safety of antibiotics are likely to be the major contributors to the disproportionate exposure to antibiotics in patients with negative cultures.^11^ Underestimation of the high sensitivity of blood cultures and overestimation of the diagnostic value of abnormal biomarkers may also contribute to overtreatment.^42^ We advocate for a factual approach taking into account that many conditions that mimic sepsis are far more common than bacterial infection. The decision-making process should be based on risk factors, clinical presentation, and diagnostic tests. In infants that are started on empirical antibiotics, the need to continue treatment should be reassessed at least on a daily basis, relying on the changes in clinical condition, microbiology results, with or without the use of biomarkers. Recent studies have shown that antibiotics started for suspected EOS can be safely discontinued within 24 h in half of the cases.^35,43^ In the future, algorithms integrating all relevant data should help clinicians in this process.

The strengths of our study derive from the large high-quality international dataset, enabling us to investigate the incidence of presumed CN-EOS and the associated antibiotic exposure across networks with different guidelines to prevent and treat suspected EOS. Our study has several limitations. As CN-EOS reflects a state of diagnostic uncertainty, there are no uniformly accepted criteria to define this condition. Based on current literature and our data, we chose a cutoff of 5 days of antibiotic treatment to define presumed CN-EOS.^9,16^ Patients with negative cultures who died before 5 days of antibiotic treatment were assigned to the CN < 5d group. This is unlikely to have affected our capacity to assess the incidence, associated antibiotic exposure and mortality of CN-EOS given the low number of these cases and since none of these deaths were related to infection. As we did not collect data on risk factors, focal infections (such as pneumonia) and biomarker levels, we could not analyze the reasons why clinicians prolonged antibiotic therapy in individual patients with negative cultures. However, prophylactic treatments accounted for a minor fraction of antibiotic exposure in our cohort.^6^ The design of our study does not allow to present incidence data stratified by gestational age which prevents us from getting insights for specific subgroups.

## Conclusions

This study sheds light on the major contribution of presumed CN-EOS to early-life antibiotic exposure. Given the uncertainty related to the diagnosis of CN-EOS, the low mortality of this condition, and its disproportionate contribution to antibiotic exposure, our study identifies CN-EOS as a major target for AMS programs.

## Supplementary information

Supplemental information

## Data Availability

The data underlying this article cannot be shared publicly due to the privacy of individuals who participated as infants in the study. The data will be shared upon reasonable request with the corresponding author.
