# Supplementary material for: Antibiotic exposure for culture-negative early-onset sepsis in late-preterm and term newborns: an international study
Source: Pediatr Res. 2024 Sep 17;97(5):1629–35. doi: 10.1038/s41390-024-03532-6 (PMC12119336; doi:10.1038/s41390-024-03532-6)
Supplement: Supplementary file 1 — Supplemental information [file 41390_2024_3532_MOESM1_ESM.pdf]

**Supplemental Information**

# Antibiotic exposure for culture-negative early-onset neonatal sepsis: an international study


**Supplemental Figure 1.** page 2

Duration of antibiotic treatment in infants with and without culture-proven early-onset sepsis.

**Supplemental Figure 2.** page 9

Incidence of culture-negative cases with a duration of treatment of at least 5 days, culture-negative cases with a duration of treatment of less than 5 days, and culture-proven early-onset sepsis.

**Supplemental Figure 3.** page 11

Incidence of culture-negative cases with a duration of treatment of at least 5 days and culture-negative cases with a duration of treatment of less than 5 days over time.

**Supplemental Figure 4.** page 13

Antibiotic exposure for culture-negative case with a duration of treatment of at least 5 days, culture-negative case with a duration of treatment of less than 5 days over time, and culture-proven early-onset sepsis.

**Supplemental Figure 1. Duration of antibiotic treatment in infants with and without culture-proven early onset sepsis.**

Histogram and density plots of the duration of antibiotic treatment for infants with culture-proven-EOS (CP-EOS) and for infants with negative cultures (No CP-EOS) in each network. Data are presented as the proportion of infants with CP-EOS and with No CP-EOS.

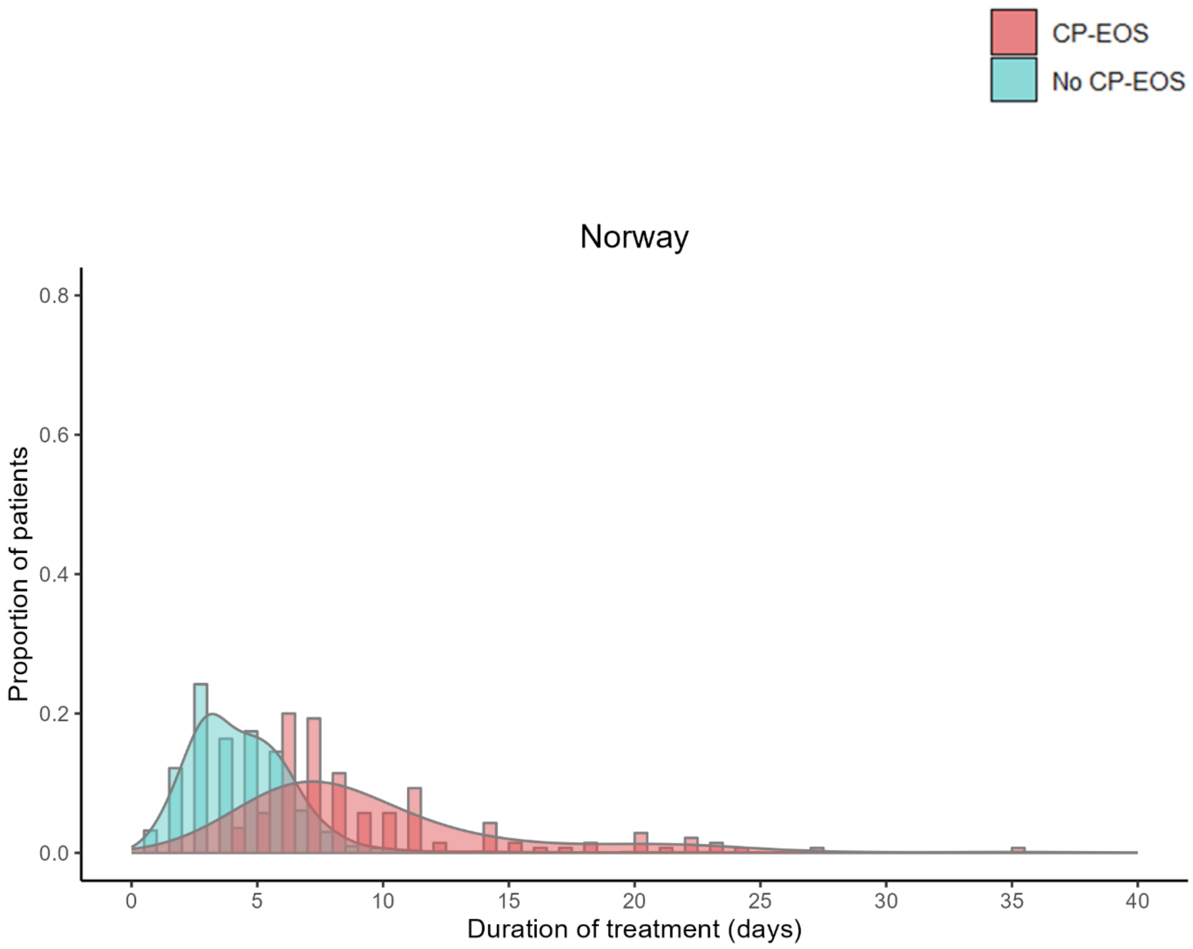

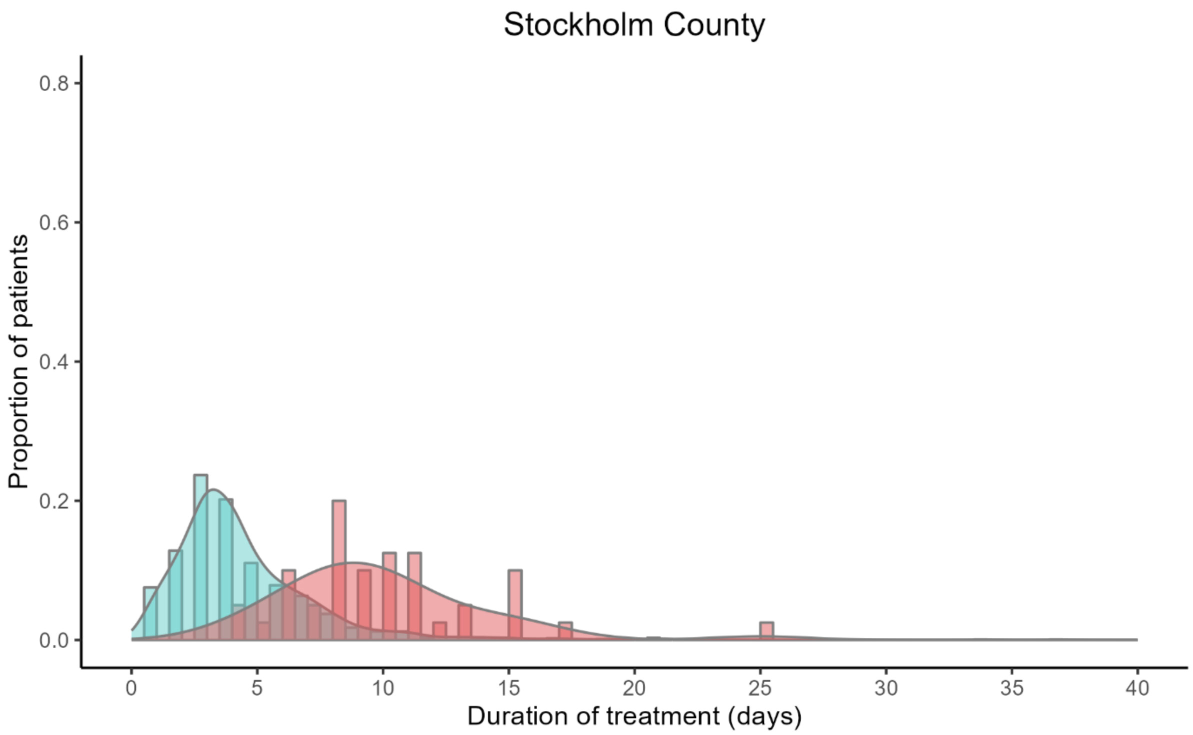
Stockholm County
Proportion of patients
0.8
0.6
0.4
0.2
0.0
0
5
10
15
20
25
30
35
40
Duration of treatment (days)

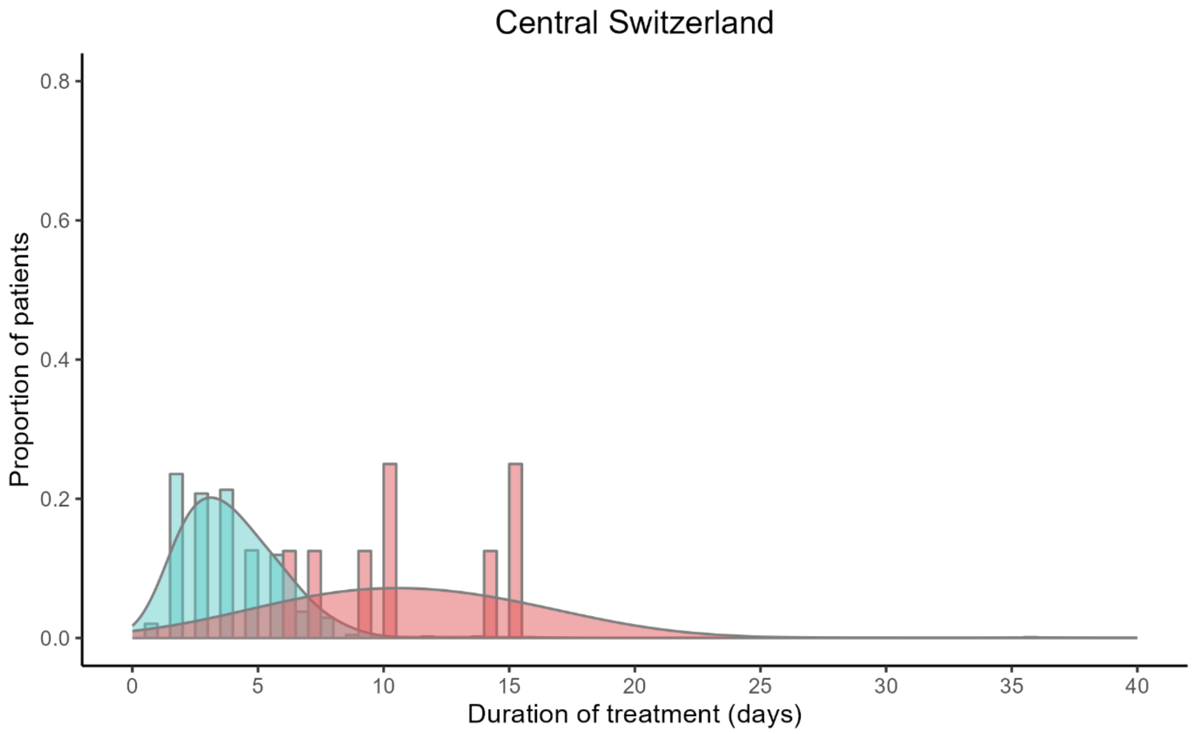
Central Switzerland
Proportion of patients
0.8
0.6
0.4
0.2
0.0
0
5
10
15
20
25
30
35
40
Duration of treatment (days)

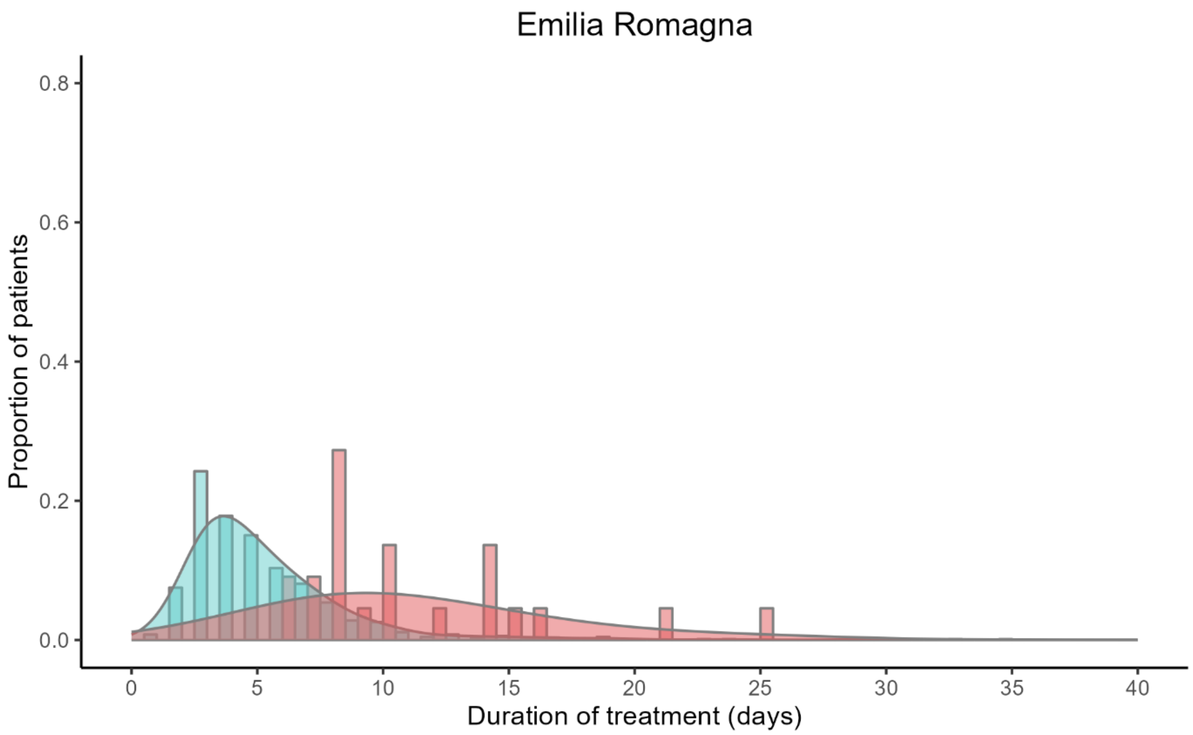
Emilia Romagna
Proportion of patients
0.0
0.2
0.4
0.6
0.8
0
5
10
15
20
25
30
35
40
Duration of treatment (days)

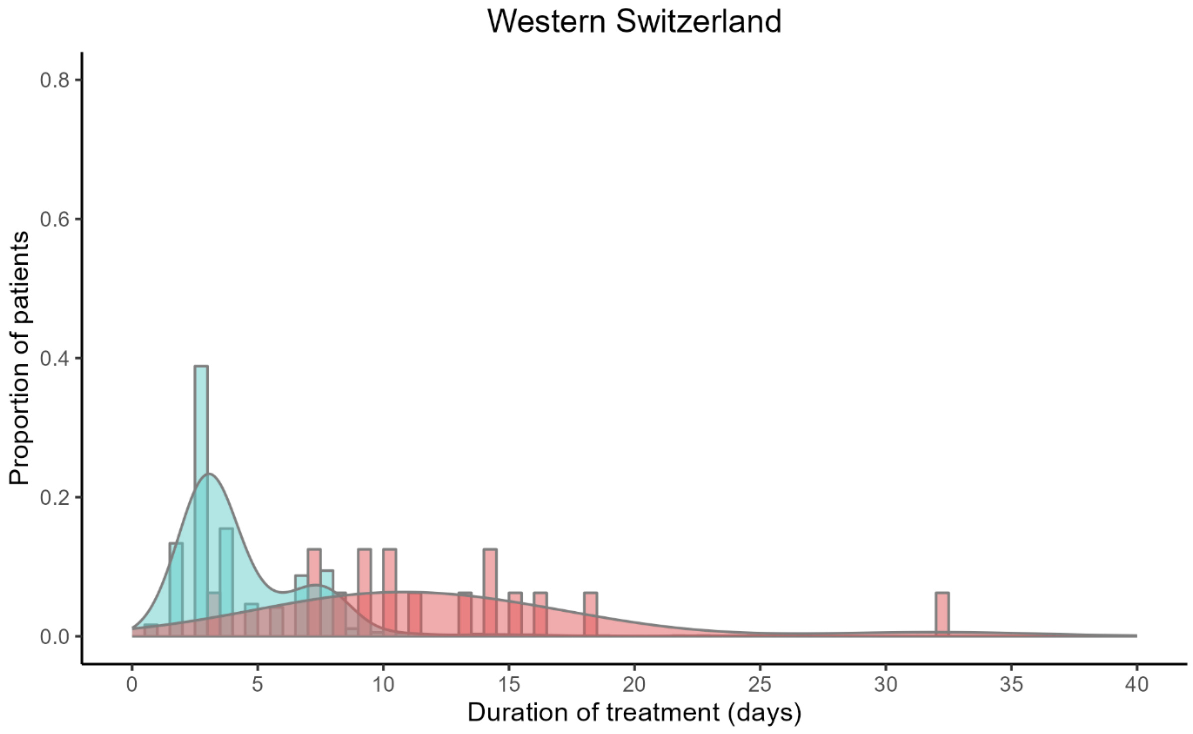
Western Switzerland
Proportion of patients
0.0
0.2
0.4
0.6
0.8
0
5
10
15
20
25
30
35
40
Duration of treatment (days)

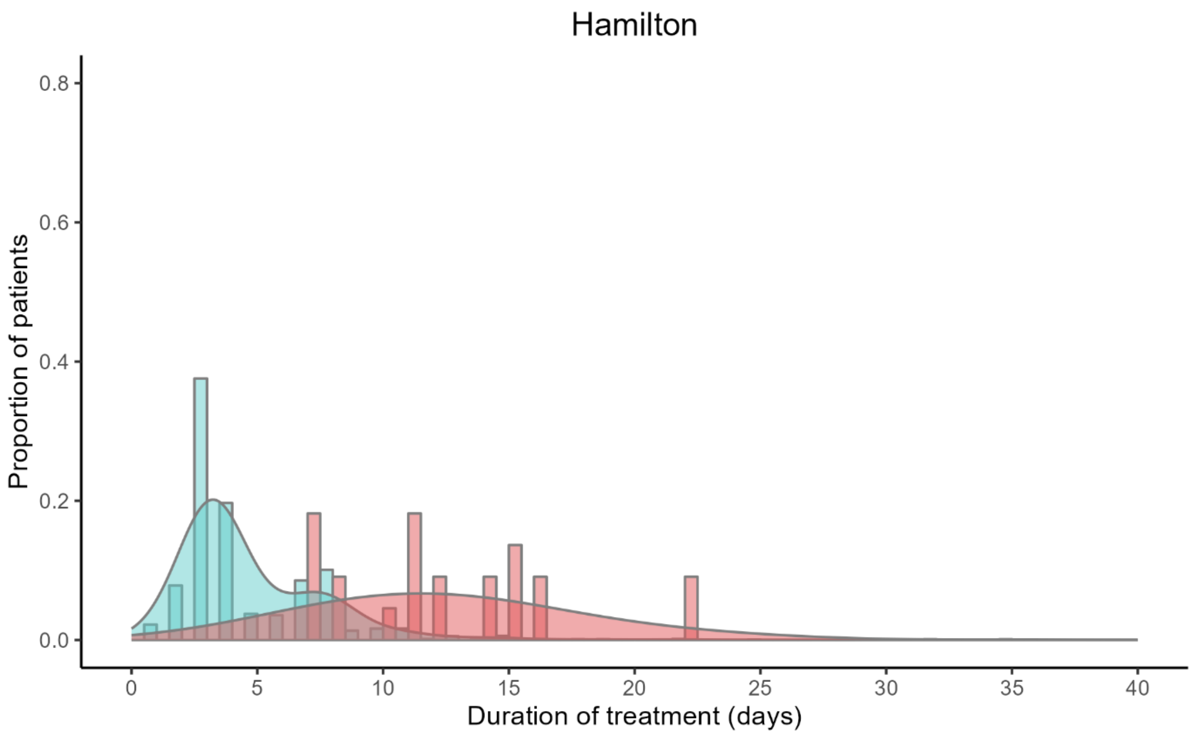
Hamilton
Proportion of patients
0.8
0.6
0.4
0.2
0.0
0
5
10
15
20
25
30
35
40
Duration of treatment (days)

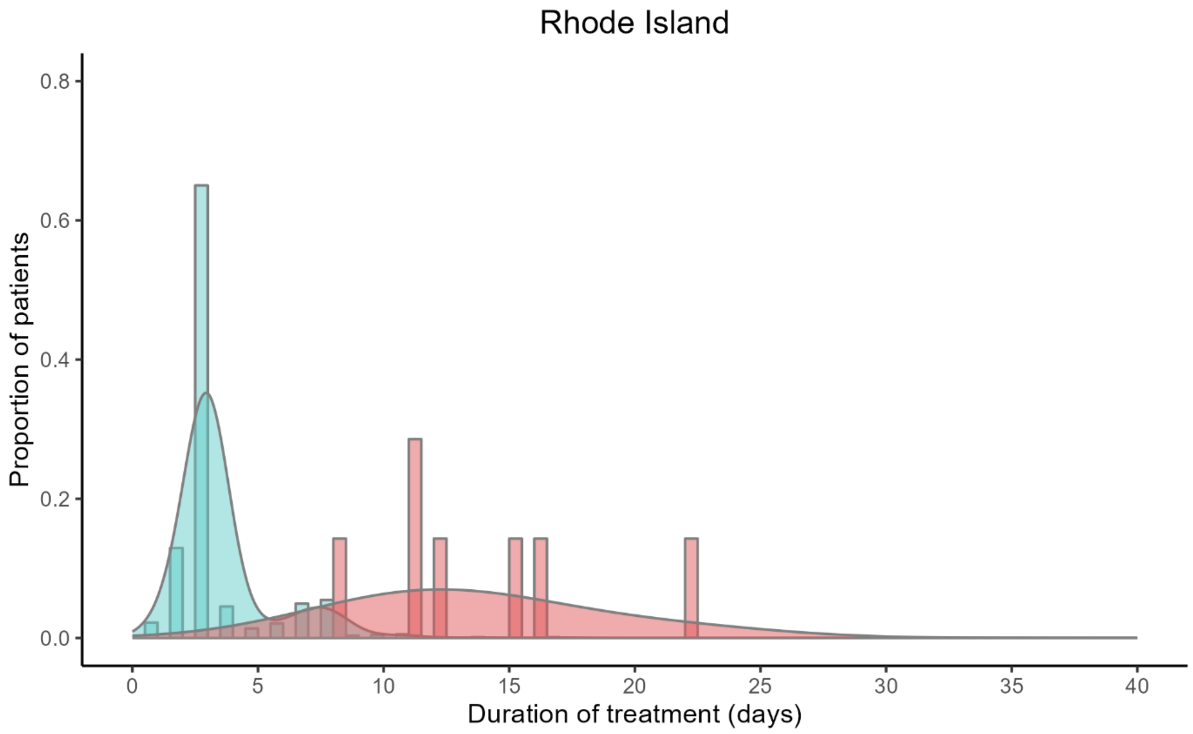
Rhode Island
Proportion of patients
0.8
0.6
0.4
0.2
0.0
0
5
10
15
20
25
30
35
40
Duration of treatment (days)

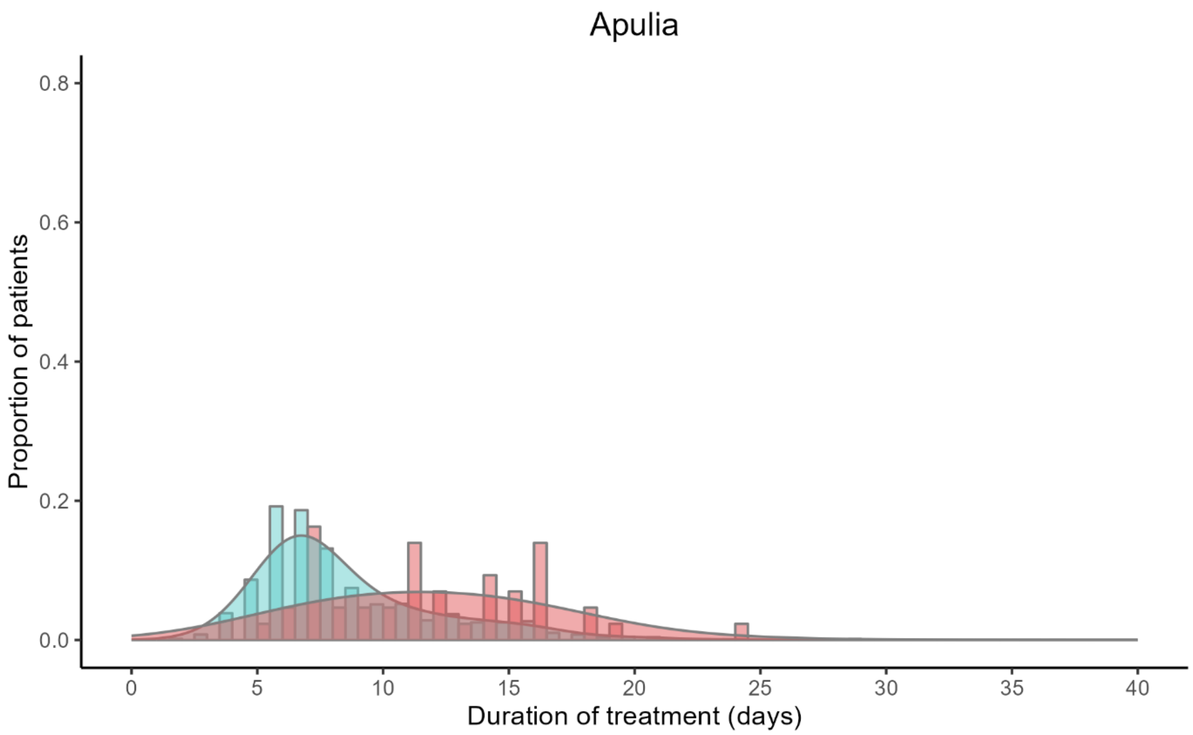
Apulia
Proportion of patients
0.0
0.2
0.4
0.6
0.8
0
5
10
15
20
25
30
35
40
Duration of treatment (days)

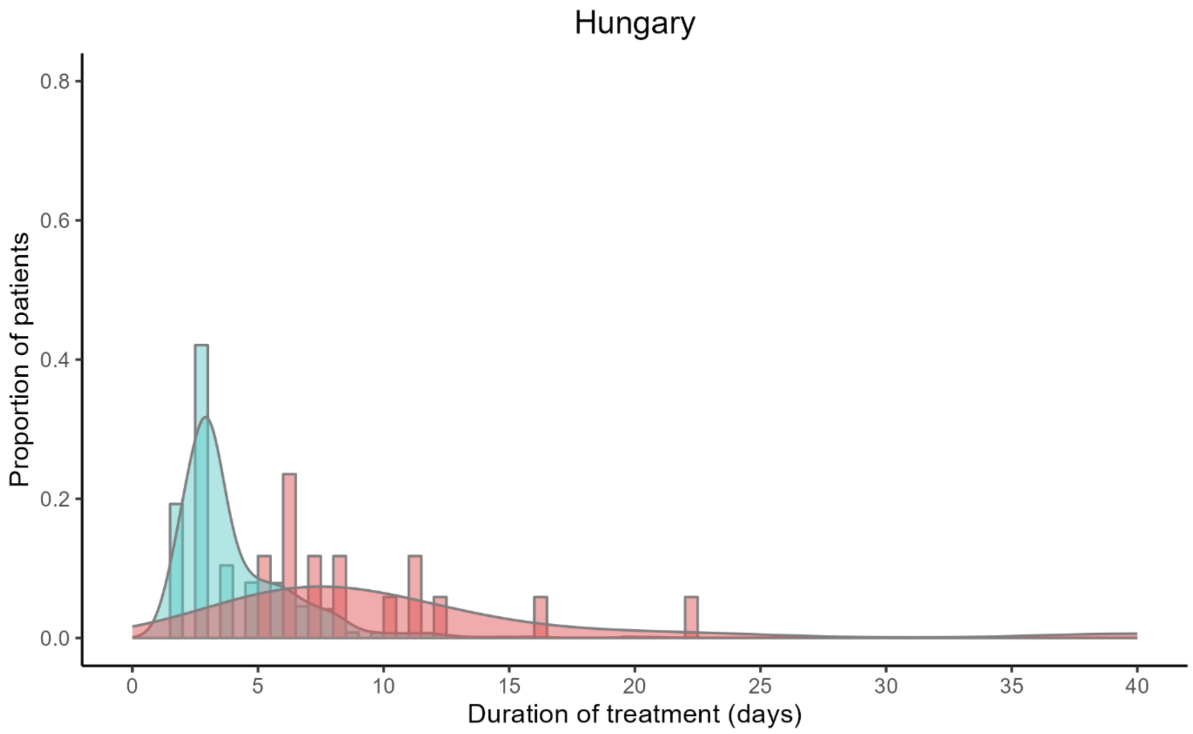
Hungary
Proportion of patients
0.0
0.2
0.4
0.6
0.8
0
5
10
15
20
25
30
35
40
Duration of treatment (days)

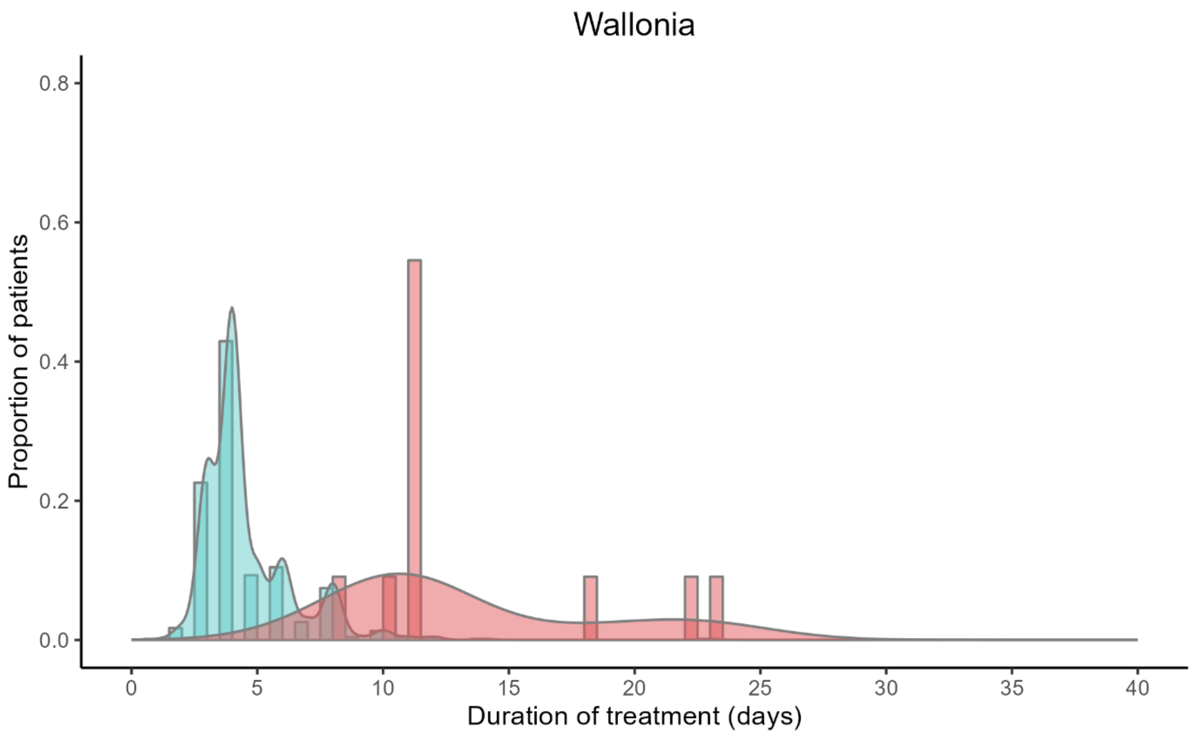
Wallonia
Proportion of patients
0.8
0.6
0.4
0.2
0.0
0
5
10
15
20
25
30
35
40
Duration of treatment (days)

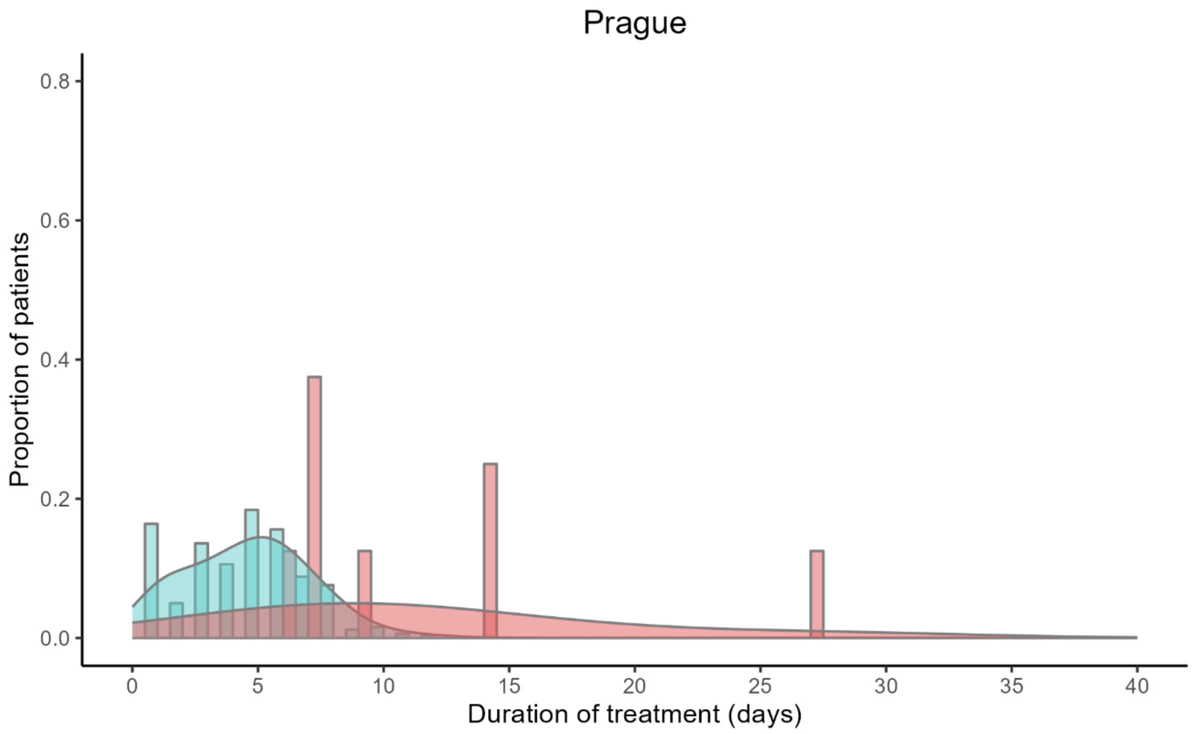
Prague
Proportion of patients
0.8
0.6
0.4
0.2
0.0
0
5
10
15
20
25
30
35
40
Duration of treatment (days)

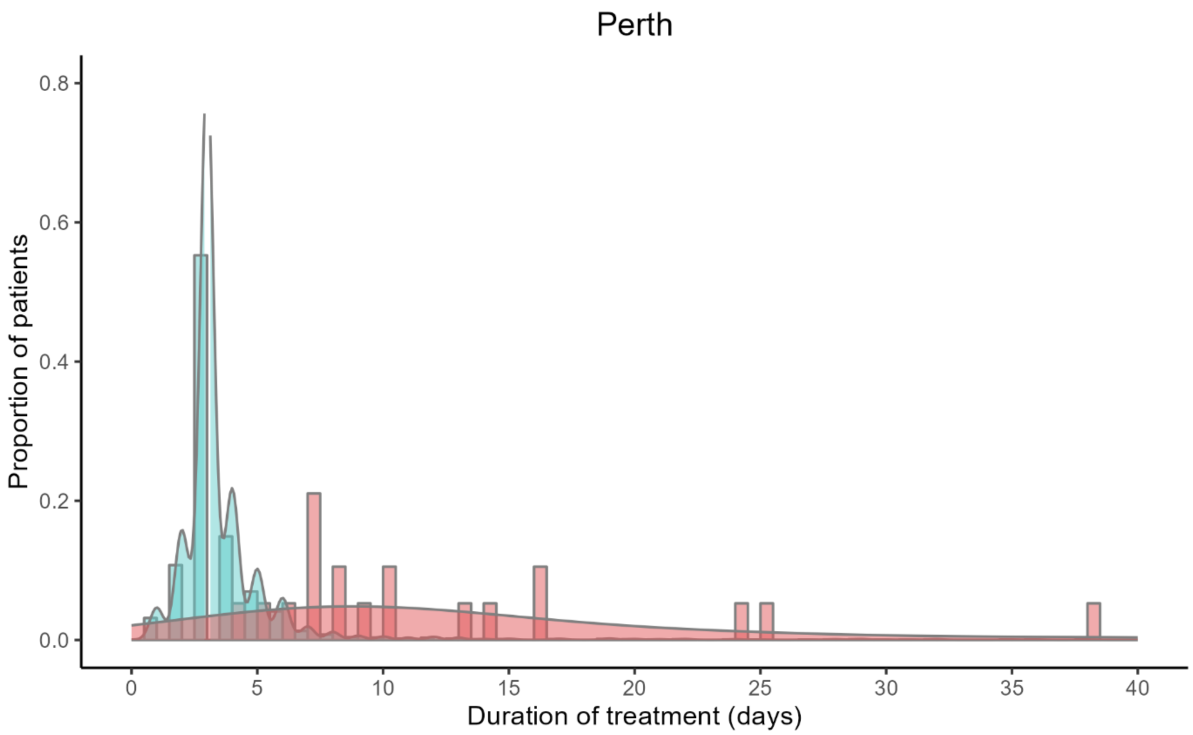
Perth
Proportion of patients
0.0
0.2
0.4
0.6
0.8
0
5
10
15
20
25
30
35
40
Duration of treatment (days)

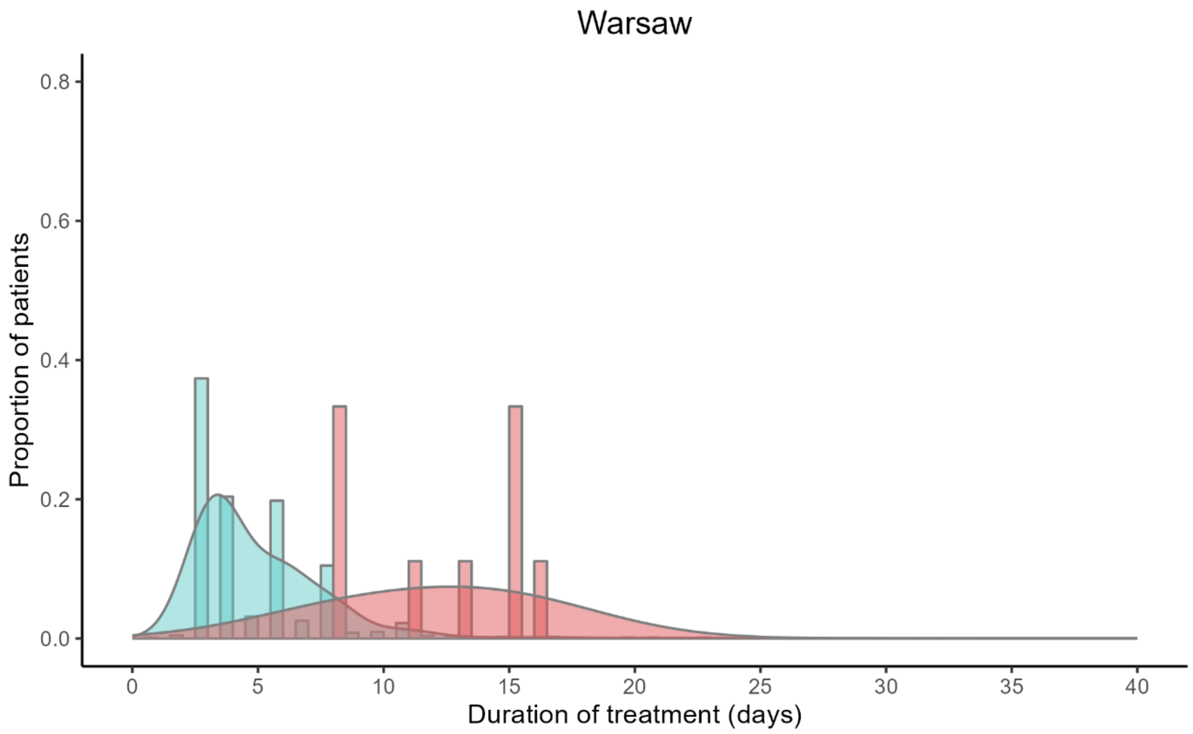
Warsaw
Proportion of patients
0.0
0.2
0.4
0.6
0.8
0
5
10
15
20
25
30
35
40
Duration of treatment (days)

**Supplemental Figure 2. Incidence of culture-negative cases with a duration of treatment of at least 5 days, culture-negative cases with a duration of treatment of less than 5 days, and culture-proven early-onset sepsis**

Incidence of culture-negative cases with a duration of treatment of at least 5 days (CN≥5d) (A), culture-negative case with a duration of treatment of less than 5 days (CN<5d) (B), and culture-proven early-onset sepsis (CP-EOS) (C) in each network. The dashed lines represent the median of the 13 networks.

**A**

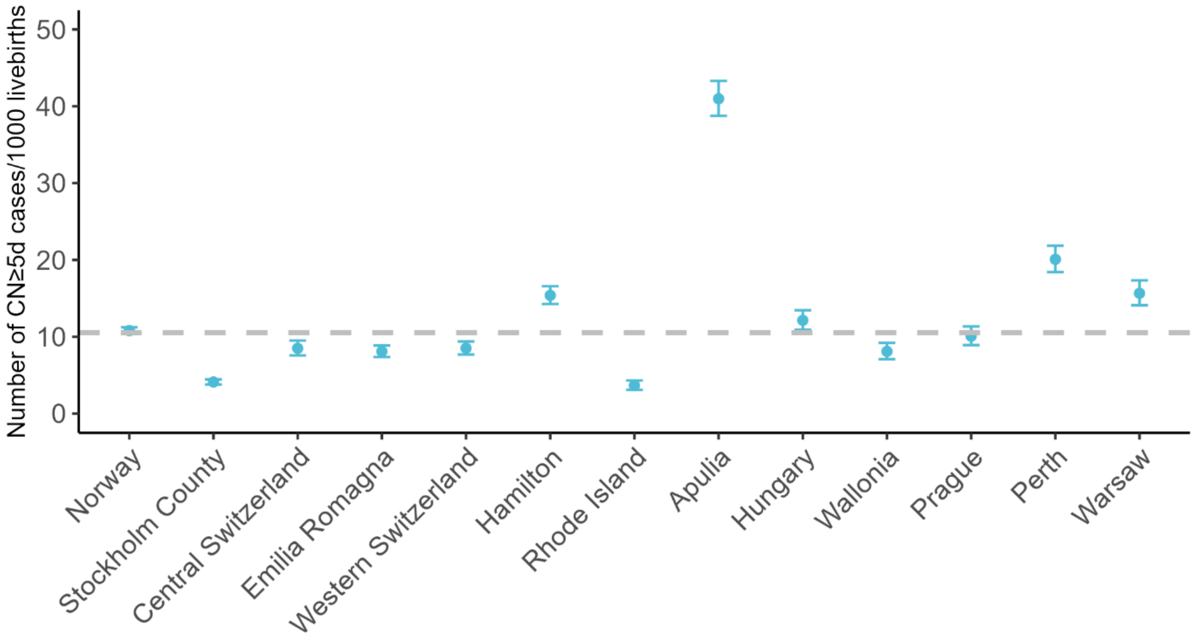

**B**

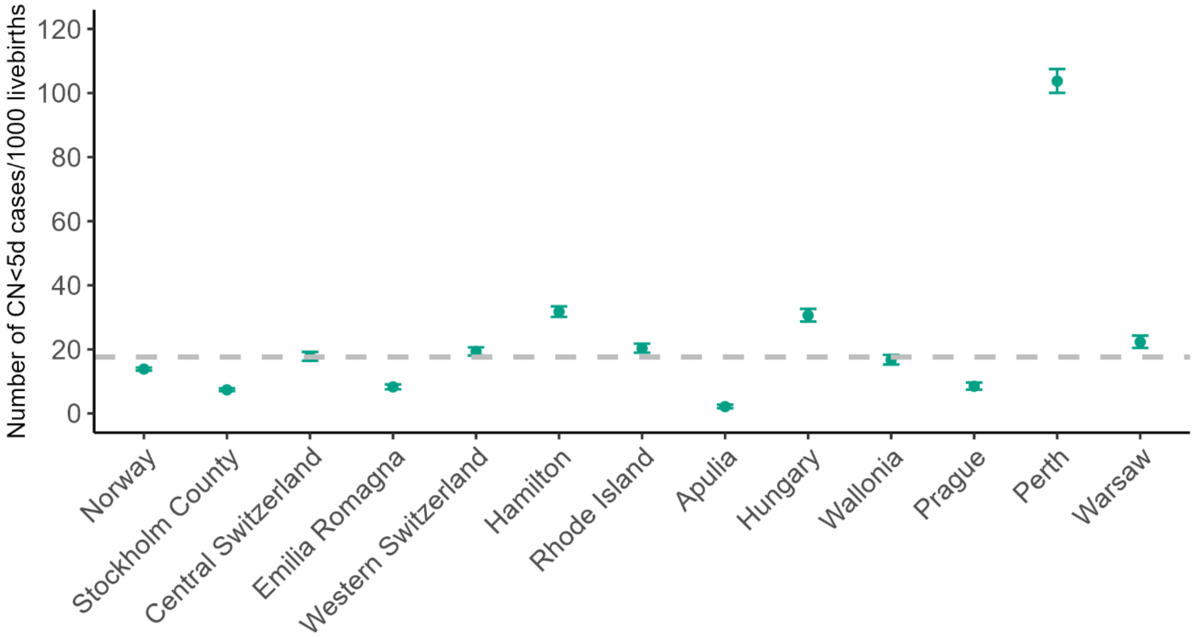
Number of CN<5d cases/1000 livebirths
120
100
80
60
40
20
0
Norway
Stockholm County
Central Switzerland
Emilia Romagna
Western Switzerland
Hamilton
Rhode Island
Apulia
Hungary
Wallonia
Prague
Perth
Warsaw

**C**

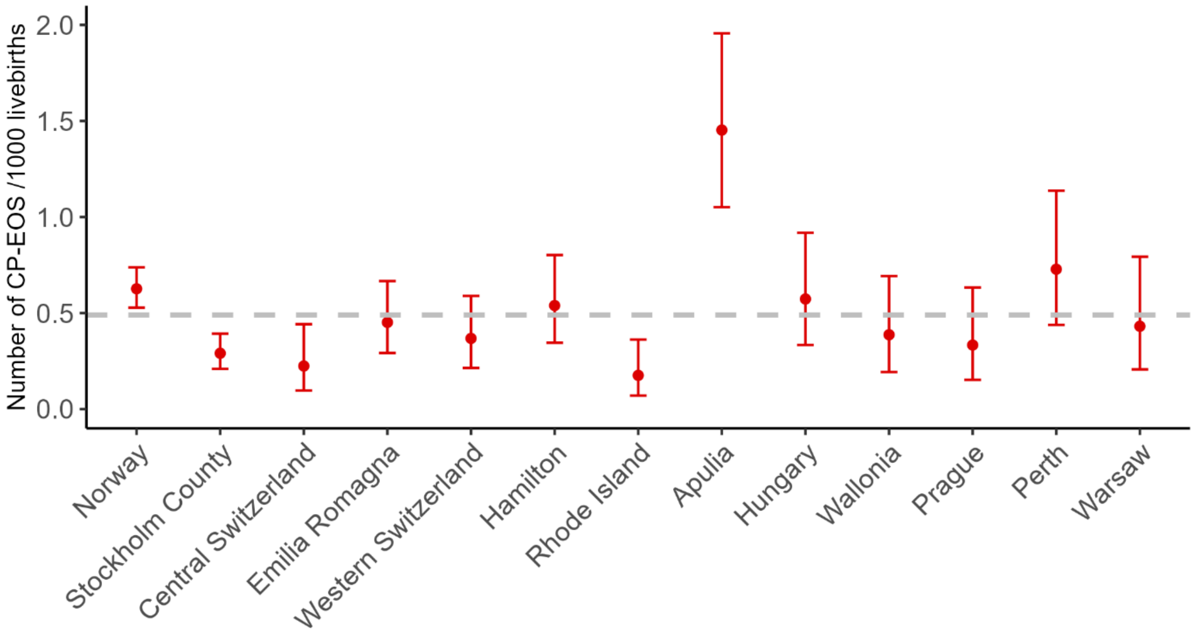
Number of CP-EOS /1000 livebirths
2.0
1.5
1.0
0.5
0.0
Norway
Stockholm County
Central Switzerland
Emilia Romagna
Western Switzerland
Hamilton
Rhode Island
Apulia
Hungary
Wallonia
Prague
Perth
Warsaw

**Supplemental Figure 3. Incidence of culture-negative cases with a duration of treatment of at least 5 days and culture-negative cases with a duration of treatment of less than 5 days over time.**

Incidence of culture-negative cases with a duration of treatment of at least 5 days (CN≥5d) by year for the 13 networks (A), and incidence of culture-negative cases with a duration of treatment of less than 5 days (CN<5d) by year for the 13 networks (B). The dashed lines represent the median of the 13 networks.

**A**

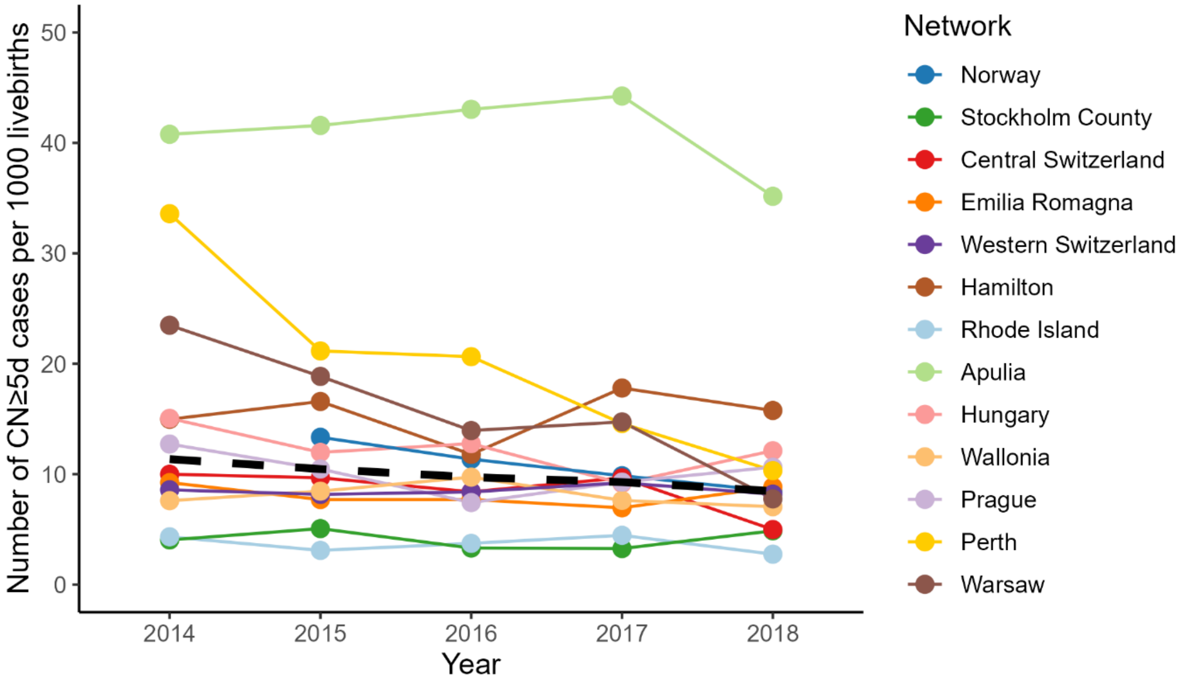

**B**

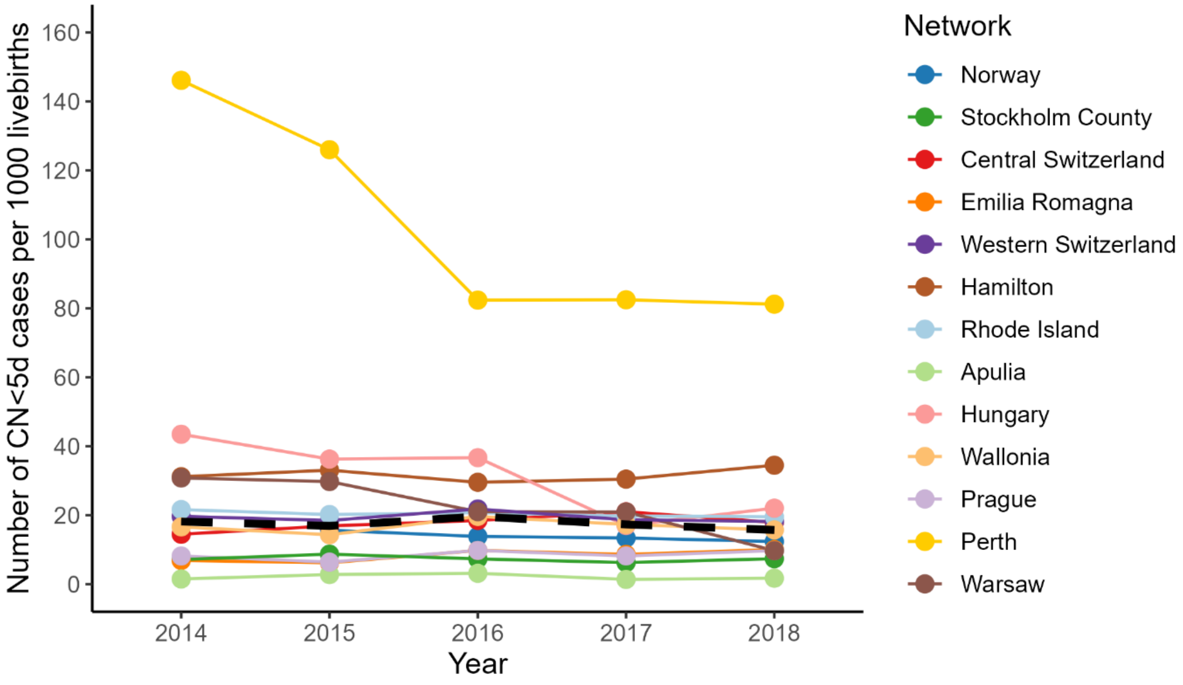

Number of CN<5d cases per 1000 livebirths
160
140
120
100
80
60
40
20
0
2014
2015
2016
2017
2018
Year
Network
Norway
Stockholm County
Central Switzerland
Emilia Romagna
Western Switzerland
Hamilton
Rhode Island
Apulia
Hungary
Wallonia
Prague
Perth
Warsaw

**Supplemental Figure 4. Antibiotic exposure for culture-negative cases with a duration of treatment of at least 5 days, culture negative treatment of less than 5 days, and culture-proven early-onset sepsis over time.**

Number of antibiotic days per 1'000 livebirths by year for each network in culture-negative cases with a duration of treatment of at least 5 days (CN≥5d) (A), culture-negative cases with a duration of treatment of less than 5 days (CN<5d) (B), and culture-proven early-onset sepsis cases (C). The dashed lines represent the median of the 13 networks.

**A**

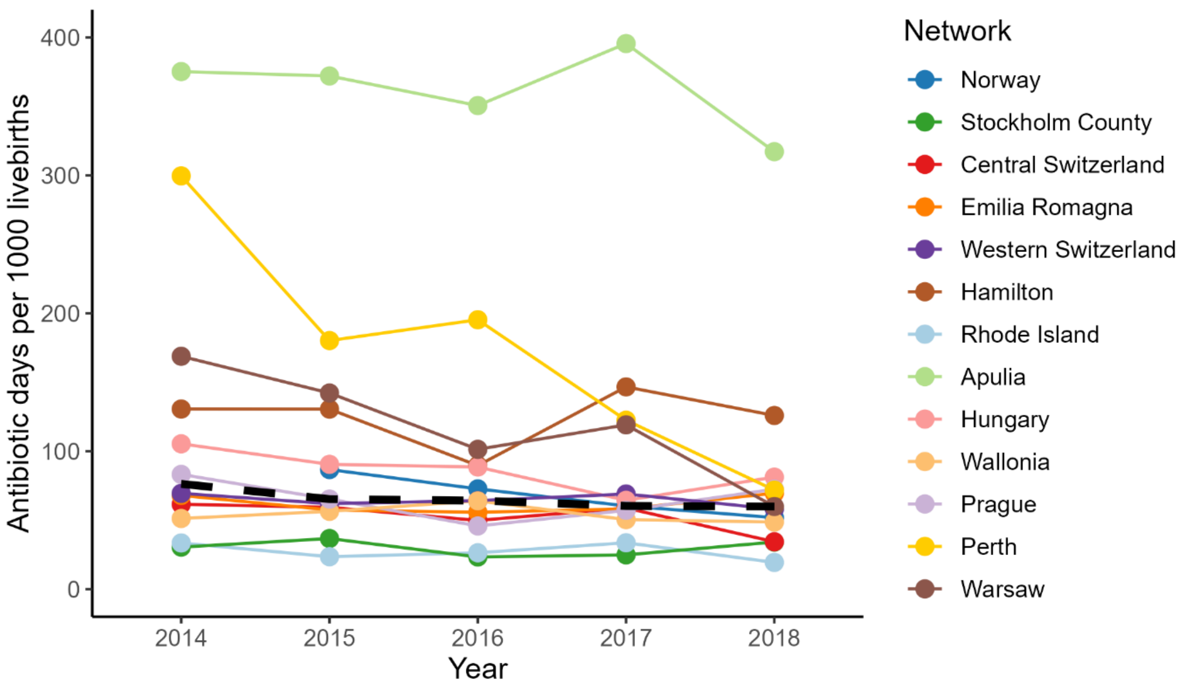

**B**

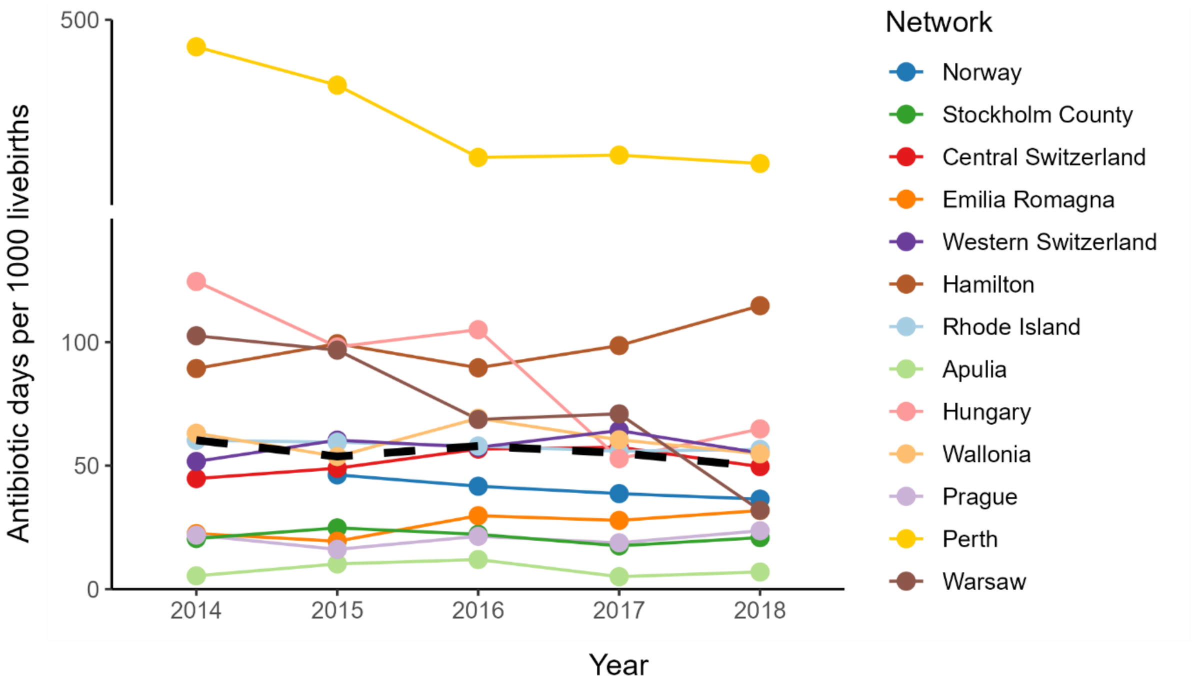

500
100
50
0
Antibiotic days per 1000 livebirths
2014
2015
2016
2017
2018
Year
Network
Norway
Stockholm County
Central Switzerland
Emilia Romagna
Western Switzerland
Hamilton
Rhode Island
Apulia
Hungary
Wallonia
Prague
Perth
Warsaw


**C**

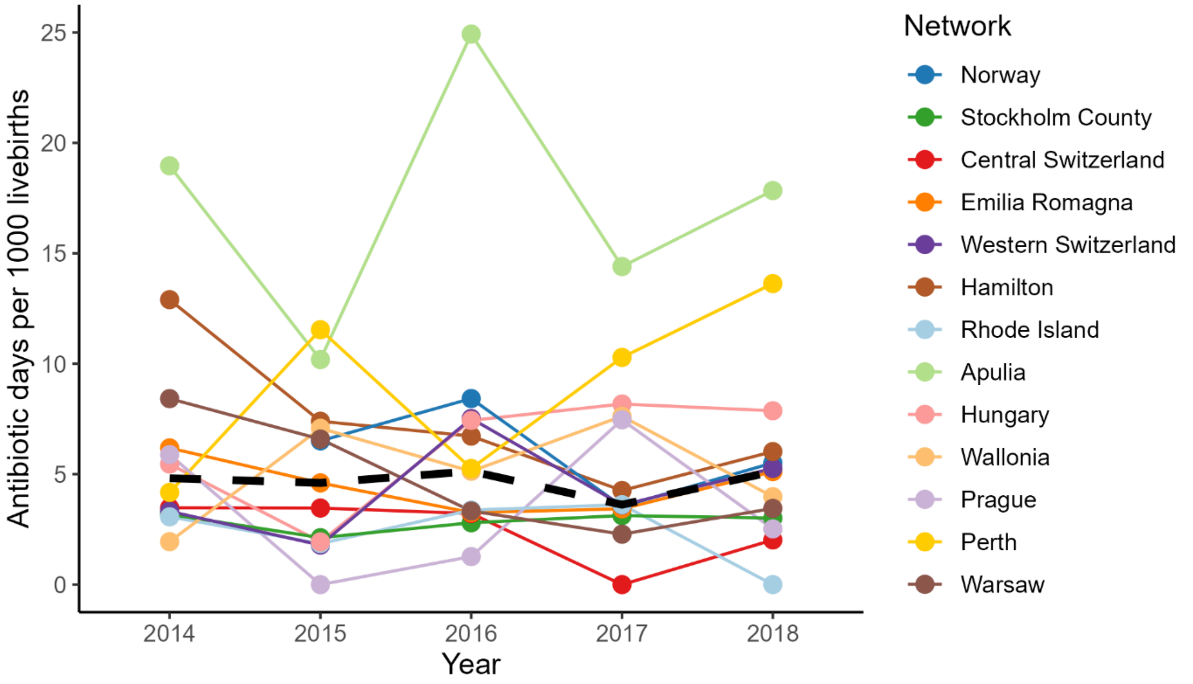

25
20
15
10
5
0
Antibiotic days per 1000 livebirths
2014
2015
2016
2017
2018
Year
Network
Norway
Stockholm County
Central Switzerland
Emilia Romagna
Western Switzerland
Hamilton
Rhode Island
Apulia
Hungary
Wallonia
Prague
Perth
Warsaw
